# Extragonadal germ cell tumors: Not just a matter of location. A review about clinical, molecular and pathological features

**DOI:** 10.1002/cam4.2195

**Published:** 2019-09-30

**Authors:** Andrea Ronchi, Immacolata Cozzolino, Marco Montella, Iacopo Panarese, Federica Zito Marino, Sabrina Rossetti, Paolo Chieffi, Marina Accardo, Gaetano Facchini, Renato Franco

**Affiliations:** ^1^ Pathology Unit Department of Mental and Physical Health and Preventive Medicine University of Campania “L. Vanvitelli” Naples Italy; ^2^ Department of Psychology University of Campania “L. Vanvitelli” Caserta Italy; ^3^ Uro‐Andrologic Oncology Unit Department of Uro‐Gynaecological Oncology Istituto Nazionale Tumori “Fondazione G. Pascale”—IRCCS Naples Italy

**Keywords:** choriocarcinoma, chromosome 12p, embryonal carcinoma, extragonadal germ cell tumors, SALL4, seminoma, SOX2, teratoma, yolk sac tumor

## Abstract

Extragonadal germ cell tumors (EGGCTs) are uncommon neoplasms, which arise in anatomical locations other than gonads. The pathogenesis of these neoplasms is still poorly understood and it is a matter of debate if they really represent extragondal primary neoplasms or rather extragondal metastasis from occult gonadal neoplasms. The actual observations suggest that EGGCTs represent a unique entity, so their biology and behavior are substantially different from gonadal counterparts. The diagnosis of EGGCTs is often challenging, and differential diagnosis is particularly wide. Nevertheless, a correct diagnosis is essential for the correct management of the patient. We summarize the state of art about EGGCTs, with particular emphasis on diagnosis and prognosis.

## Graphical abstract

Extragonadal germ cell tumors (EGGCTs) are uncommon neoplasms with peculiar molecular and pathological features; the diagnosis of these neoplasms may be challenging, due to wide differential diagnosis depending to the variable anatomical location. The authors summarize the state of art about EGGCTs, with particular emphasis on diagnosis and prognosis.

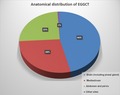

## 1 INTRODUCTION

Extragonadal germ cell tumors (EGGCTs) are a heterogeneous group of tumors of neoplastic germ cells arising from extragonadal anatomical locations, without evidence of gonadal primary tumors. EGGCTs include seminomatous tumors, including only classical seminoma, and nonseminomatous tumors (NST), including embryonal carcinoma (EC), teratoma (mature or immature), yolk sac carcinoma (YST) and choriocarcinoma. EGGCTs constituted by two or more histotypes are referred to as *Mixed Germ Cell Tumors*. Although EGGCTs are morphologically identical to their gonadal counterparts, current knowledge about these neoplasms shows that they represent a unique entity and their biology is substantially different from their gonadal counterparts.

## 2 EPIDEMIOLOGY

**Figure 1:**
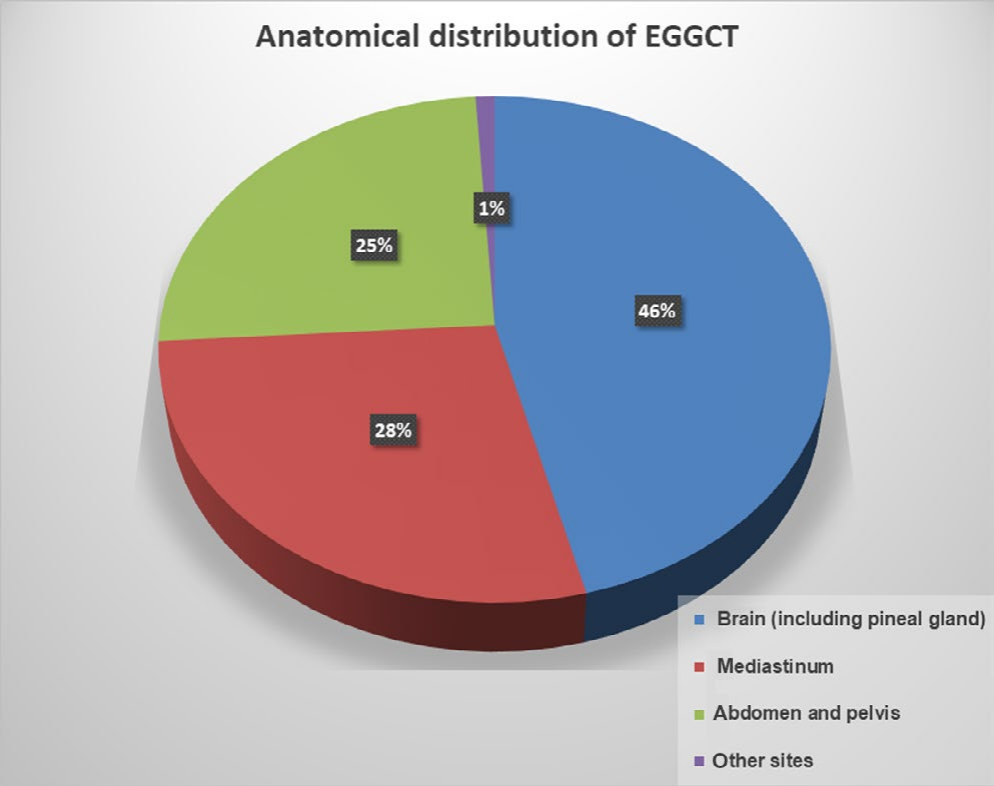
Anatomical distribution of extragonadal germ cell tumors (EGGCTs)

EGGCTs are uncommon neoplasms. Stang et al. have recently reported epidemiologic data related to the incidence and survival of EGGCTs in the US from 1973 to 2007.^1^ According to this series, the overall incidence ranges from 1.8 to 3.4/1 million; females are less commonly affected than males and the highest incidence has been observed in white males (56.3/1 million).^1^ EGGCTs may localize in almost every structure along the midline of the body, from the brain to the coccyx. However, the most common anatomical locations are represented by the mediastinum, retroperitoneum, and brain,^1^ while the pineal gland and sacrococcygeal area are fewer common locations.^2^, ^3^ Isolated cases have been reported in bladder,^4^ prostate,^5^ paratesticular adnexa,^6^ vulva,^7^ placenta, pelvis, uterus,^8^ kidney,^9^ nasal sinuses^10^ and other sites. The anatomical distribution of EGGCT is summarized in Figure 1. Epidemiology of EGGCTs varies widely in age and gender groups of patients. Particularly, Teratoma is the most common EGGCT in prepuberal age regardless of the gender. It could be observed as pure or mixed tumor mainly associated to YST or, more rarely, to EC. In prepuberal patients, the most common anatomical sites include the sacrococcygeal area, intracranial, mediastinum, head and neck and peritoneum.^11^, ^12^ Sacrococcygeal teratoma is one of the most common congenital tumors, with a prevalence of 1/27 000 live births and a male‐to‐female ratio of 1:4.^1^ Mediastinal masses are rarely EGGCTs, representing up to 16% of all mediastinal neoplasms in adults and up to 19%‐25% in population younger than 18 years.^13^ It is well recognized that recurrences or metastasis from neonatal pure teratomas can acquire a different phenotype including mixed teratoma‐YST or even pure YST.^14^ These two different possibilities could be related to an inadequate sampling of the surgical sample, but also to a real “progression” of the neoplasm. It seems that in older patients, sacrococcygeal location and presence of histological immaturity are frequently associated with a higher incidence of mixed teratoma‐YST.^11^ In addition, metastases from a mixed EGGCT are frequently constituted by teratomatous component. The teratomatous metastasis is also observed sometimes even in case of pure EGGCT other than teratoma; in these clinical settings, an unrecognized mixed component or a posttherapy differentiation is supposed.^15^ In adults, the epidemiology of EGGCTs depends on sex. Teratoma is by far the most common histotype in females, representing up to 90% of all EGGCTs. Teratoma, seminoma, YST and mixed tumors are quite equally represented in males.^16^ Seminoma is extremely rare before puberty and particularly in children less than 10 years of age.^17^

## 3 ETIOPATHOGENESIS

The exact etiopathogenesis of EGGCTs is not clearly demonstrated. Indeed, we still refer to the classical hypothesis of the “embryonic” origin of such neoplasms from germ cell precursors erroneously arrested in midline migration during the embryogenesis.^18^ Primordial GC originating from the proximal epiblast normally migrate to the genital ridge following the body midline. Particularly the thymus could be a preferential site for primordial GC arrest, because of high expression of KIT ligands, notoriously involved in primordial GC proliferation.^19^ Although this theory is widely accepted, some Authors proposed alternative etiopathogenetic explanations. McKenney et al. argued that EGGCTs could represent metastases developed from an undiagnosed or regressed (“burned out”) primary GC tumors in the gonads.^20^ This theory seems to be corroborated by some observations. Indeed, the possibility of various degrees of regression in cases of gonadal GCTs has been well documented. Although this usually presents as focal areas occupied by fibrous scars at the periphery of the neoplasm, it can be diffuse and affect the whole neoplasm.^21^ Furthermore, survival rates are lower in EGGCTs than in primary gonadal GCTs, as expected by metastatic disease.

Genetic studies recently allowed a better understanding of the etiopathogenesis of EGGCTs. Aneuplody and Chromosome 12 abnormalities are the most frequent genetic aberrations observed in postpuberal GCTs. In particular, Chromosome 12 abnormalities, most often resulting in 12p overexpression, have been demonstrated in 96% of mediastinal seminoma.^22^ Limited data are actually known about the genetic alteration of non‐seminomatous GCTs. An increased number of chromosome 12p copies, mainly as isochromosome i(12p), have been most commonly described.^23^ i(12p) is considered highly specific for the germ cell origin of postpuberal neoplasm, but not in prepuberal gonadal neoplasm.^24^, ^25^ While the genetic alterations of gonadal GCTs have been better described, 12p status of EGGCTs are yet to be adequately studied. Recently Gurda et al. described the absence of chromosome 12p alterations, including i(12p), in 11 prepubertal and five postpubertal mature sacrococcygeal teratomas and immature prepubertal sacrococcygeal teratomas.^26^ These data suggest that i(12p) could be less important in the pathogenesis of EGGCTs when compared with postpuberal gonadal counterparts, underlining a higher proximity to prepuberal gonadal neoplasms. Some genetic syndromes present increased incidence of EGGCTs, including Klinefelter, Down and Li‐Fraumeni syndromes.^27^ The recurrent amplification of specific chromosome arms, reciprocal deletions and K‐RAS mutations seem to be the most common primary somatic features of GCTs.^28^, ^29^ A recent study demonstrated genomic amplification of SOX2, supporting the theory that SOX2 has a relevant oncogenic role in the etiopathogenesis of EC.^30^

## 4 HISTOPATHOLOGY

**Figure 2:**
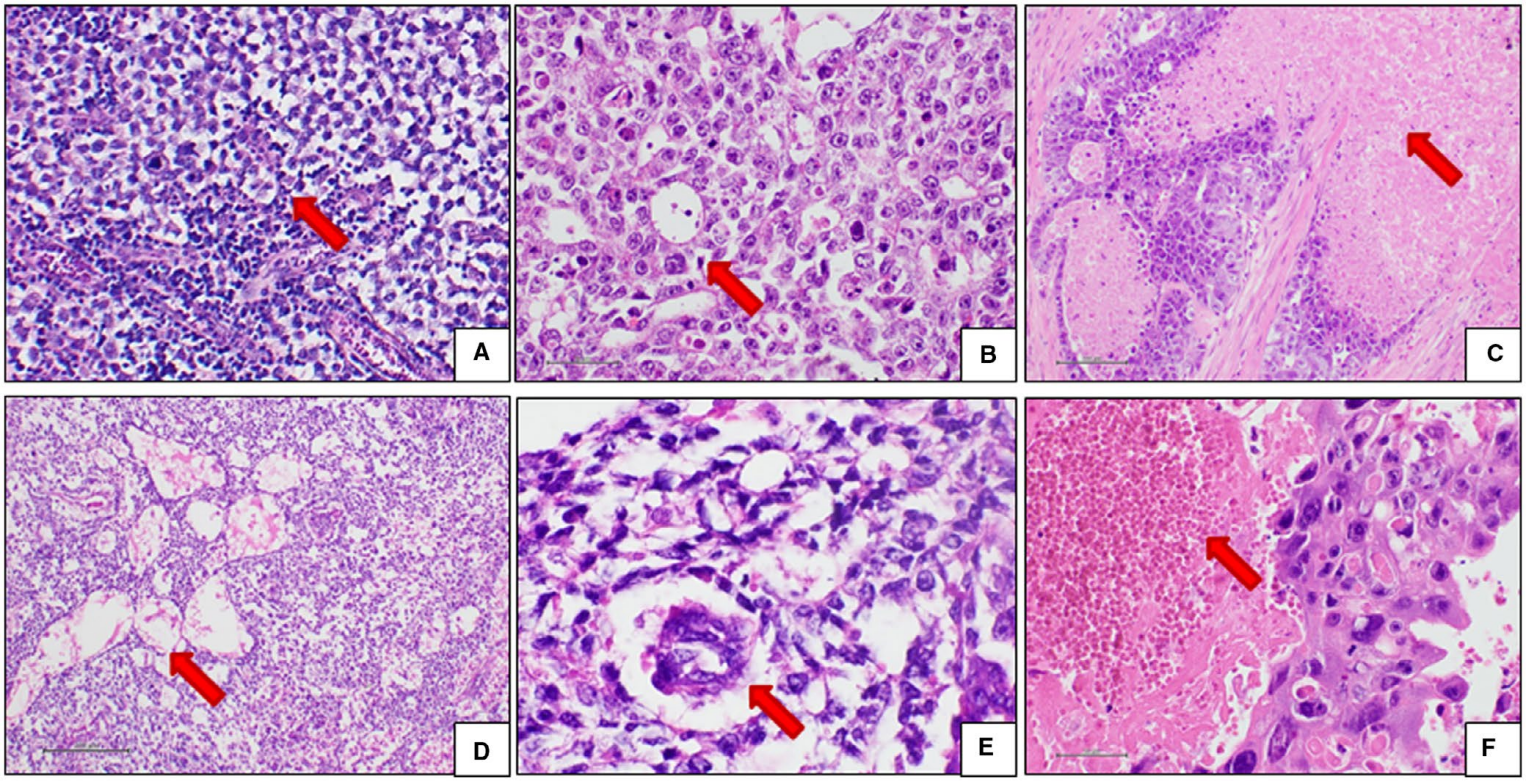
Histological features of EGGCTs. A, Seminoma. The neoplastic cells are arranged in solid lobules (red arrow) separated by thin fibrous septa containing lymphocytes. The cells are large‐sized, with abundant clear cytoplasm, roundish and relatively regular nucleus and prominent nucleolus. B, EC. The neoplastic cells are quite similar to seminoma cells, but the microscopic appearance is more variable. In this case, the architectural pattern is glandular (red arrow) and solid, and the neoplastic cells are more pleomorphic and atypical. C, EC. Coagulative necrosis (red arrow) is a diagnostic clue of EC. Attention must be paid to differentiating real coagulative necrosis from ischemic necrosis, a possible event in large or traumatized seminomas. D, YST. Relatively bland neoplastic cells arranged in the typical cystic architectural pattern (red arrow). E, YST. Schiller‐Duval bodies (red arrow) are a diagnostic clue, but they are present in about 50% of cases. F, Choriocarcinoma. The neoplastic cells are very large in size, with abundant slightly eosinophilic cytoplasm and atypical nuclei. Large hemorrhages are typically seen (red arrow). EC, embryonal carcinoma; EGGCTs, extragonadal germ cell tumors; YST, yolk sac tumor

**Figure 3:**
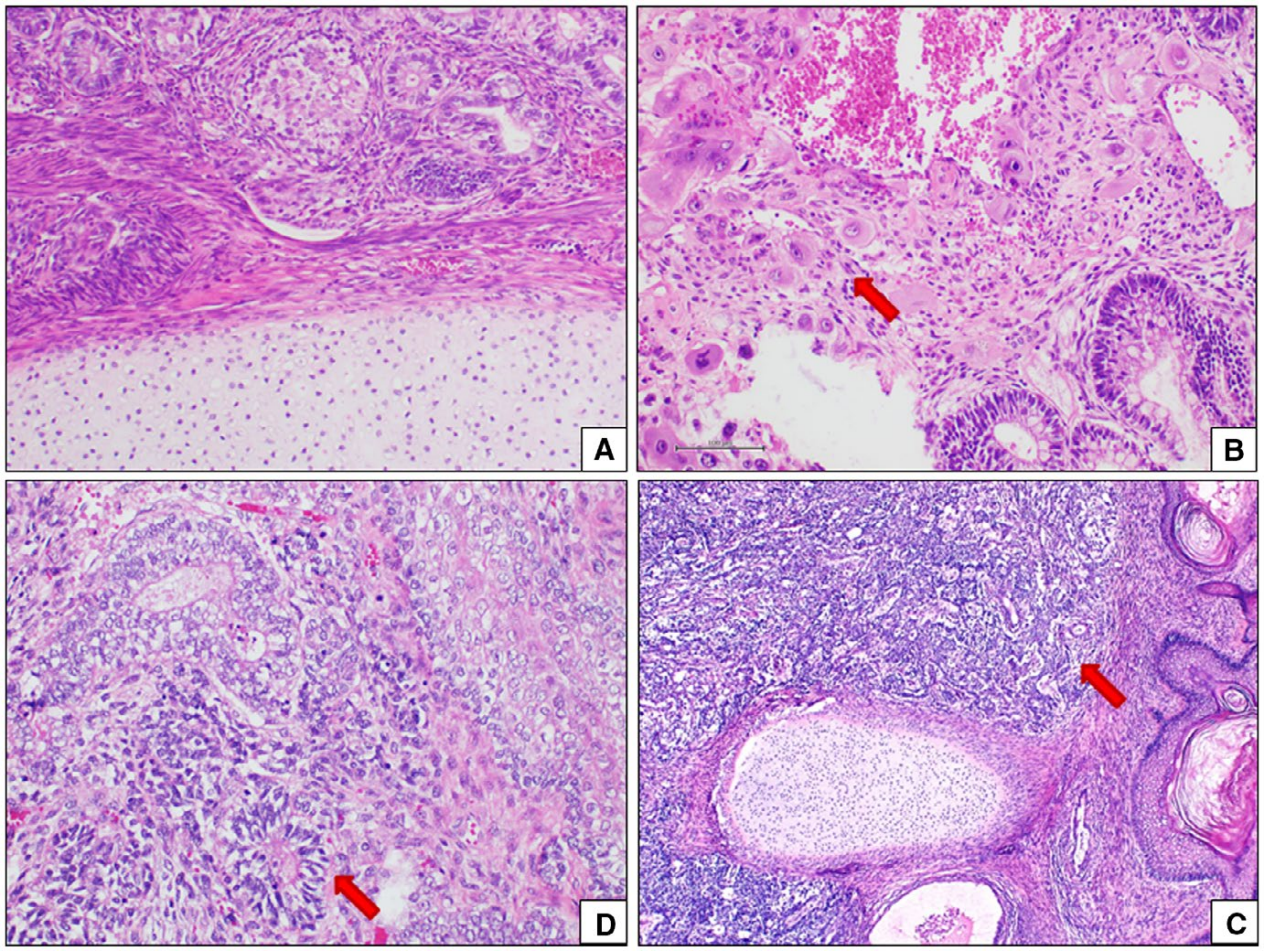
Histological features of mixed EGGCTs. Teratoma is characterizedby the coexistence in the same neoplasm of different mature or immature tissues (A). Mixed EGGCT often include a teratoma component (B: teratoma and chociocarcinoma (red arrow); (C) teratoma and EC (red arrow); (D) teratoma and YST (red arrow). EC, embryonal carcinoma; EGGCTs, extragonadal germ cell tumors; YST, yolk sac tumor

Morphological and immunohistochemical features of EGGCTs are the same as observed in the gonadal counterparts. Solid somatic malignancies can develop in the context of an EGGCT, mainly in pure teratoma or mixed EGGCT in adult males.^31^ Sarcomas (embryonal rhabdomyosarcoma, leiomyosarcoma, angiosarcoma and neuroblastoma) and adenocarcinoma are the most commonly reported neoplasms in this clinic‐pathological setting.^31^ The histological features of EGGCTs are shown in Figures 2 and 3.

### 4.1 Seminoma

Macroscopically, seminoma is an expansive, lobulated mass with a tan‐gray cut surface. The mass ranges from 1 to 20 centimeters.^32^ Histologically, seminoma shows a lobular architecture with neoplastic nodules delimitated by incomplete thin fibrous septa. The neoplastic cells are large, with clear to lightly eosinophilic cytoplasm and regular nucleus with a prominent nucleolus. A lymphoid infiltrate constituted by small mature lymphocytes is commonly present, mostly in the thickness of the fibrous septa. A granulomatous infiltrate with multinucleated giant cells and syncytiotrophoblastic cells can be present.

### 4.2 EC

Macroscopically, EC is often a large mass when diagnosed, with infiltration features of the surrounding tissues. The cut surface generally shows hemorrhagic areas and necrosis. The histological architecture is variable and more often solid, but “epithelial” arrangements are possible, including glandular and papillary structures. The cells are large‐sized, with large atypical roundish nucleolated nuclei. Cytoplasmic features are quite variable including amphophilic, basophilic, eosinophilic or clear appearances. Coagulative necrosis, “epithelial” arrangements and nuclear atypia are the most important morphological features useful for differential diagnosis with seminoma.

### 4.3 YST

Macroscopically, YST is a large mass with whitish cut surface, frequently characterized by hemorrhagic, mixoid and necrotic areas. Histologically, several possible architectural patterns are possible, including reticular/microcystic, psudopapillary/endodermal sinus, myxomatous, hepatoid, and glandular and solid patterns. The reticular or microcystic pattern is more frequently observed and is characterized by irregular channels circumscribing small cystic spaces organized in a loose network. In about 50% of YSTs, Schiller‐Duval bodies are found. The neoplastic cells are medium to large‐sized, with evident cytoplasm, roundish nucleus and prominent nucleolus. Also in YST, some scattered syncytiotrophoblastic cells could be observed.

### 4.4 Teratoma

Macroscopically, a teratoma usually appears as a well‐demarcated and encapsulated mass with a variegated cut surface, which comprises soft or fleshy areas, cystic areas containing keratinaceous debris or hair shaft, mucoid or serous material. Teeth or bone can be present. Histologically, a teratoma is composed of several somatic tissues, with various degrees of maturation, arranged in a disorganized distribution. A teratoma is distinct in mature and immature forms, depending on the resemblance to adult or fetal tissues. In immature teratoma, neuroectodermal tissue constituting tubules and rosettes is the most common fetal‐appearing tissue observed. Rarely other fetal tissues including primitive mesenchymal tissue, cartilage, bone, rhabdomyoblasts are observed.

### 4.5 Choriocarcinoma

Macroscopically, choriocarcinoma is an extensively hemorrhagic mass, infiltrating the adjacent structures at the time of the diagnosis. Histologically, choriocarcinoma is composed by syncytiotrophoblastic and cytotrophoblastic cells. The former are giant multinucleated cells. The latter are mononuclear cells with eosinophilic cytoplasm, roundish nuclei and prominent nucleoli. Large hemorrhagic areas, dense vascularity and prominent cellular atypia are distinctive features.

## 5 CYTOPATHOLOGY

**Figure 4:**
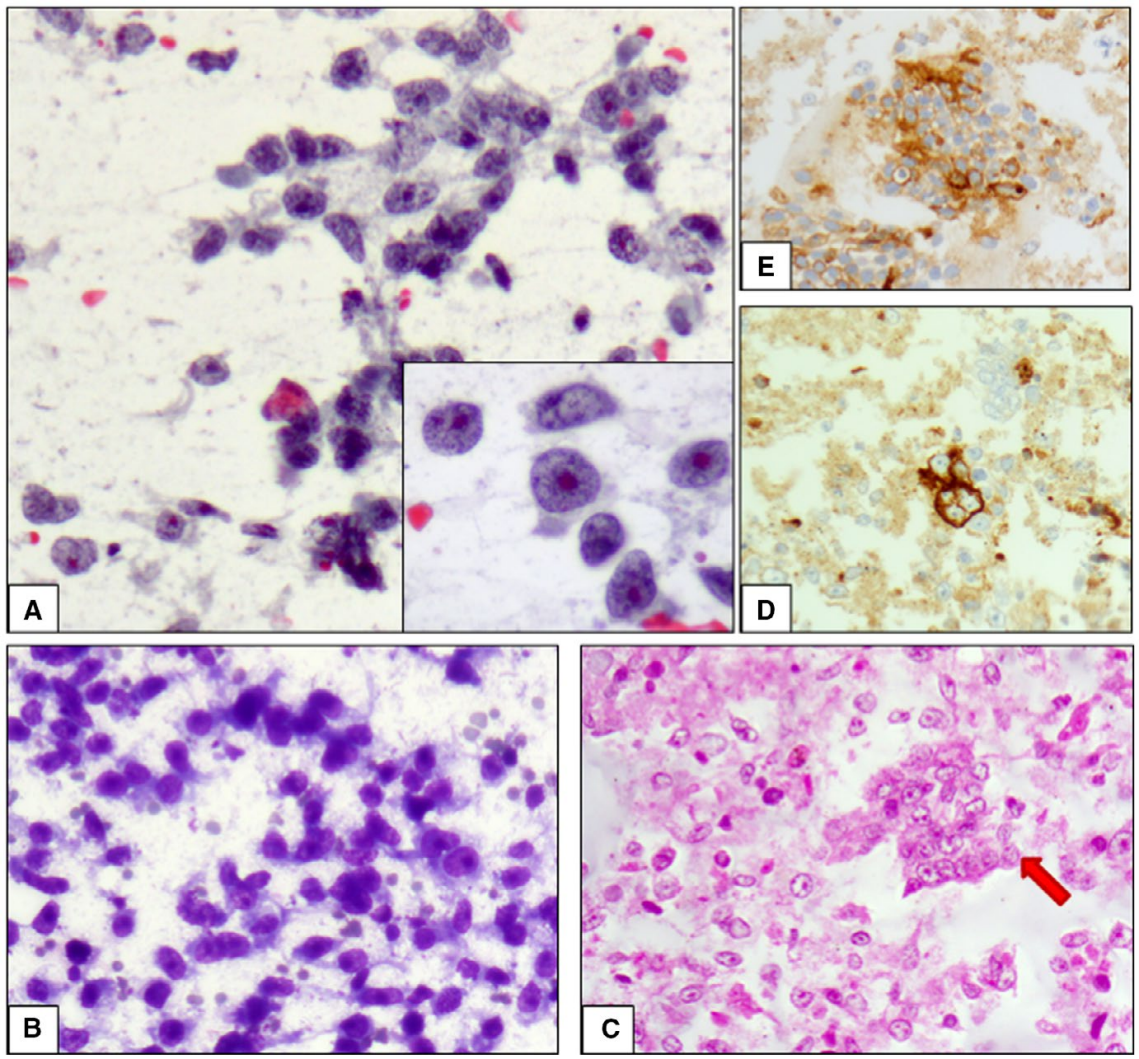
Cytological features of seminoma. A, Papanicolaou‐stained smears of seminoma are characterized by a dissociated cell population and few loose small clusters. The cells show large vesicular nuclei and prominent nucleoli and chromatin clumping (Inset). B, May‐Grümwald‐Giemsa stained smears show the characteristic tigroid (stripped) background. C, The realization of a cell block is needful and allows the evaluation of architectural disposition of neoplastic cells and the realization of immunohistochemical tests. In this case, neoplastic cells are organized in single elements and small solid fragments (red arrow), and show positivity for CD117 (D) and PLAP (E)

The diagnosis is often performed on an incisional bioptic or Fine Needle Aspiration Cytology (FNAC) sample. US‐ or CT‐guided FNAC may be a valid diagnostic alternative to biopsy for an accurate, rapid and reliable diagnosis of EGGCT. In particular, differentiation of EGGCTs in seminoma and nonseminoma tumors is possible also on cytological samples. FNAC smears with a homomorphous and dissociated cell population and few loose small clusters characterize seminoma. The cells are mononucleated and show large vesicular nuclei with frequent membrane irregularities. The nuclei show irregularly distributed chromatin. Chromatin clumping, parachromatin clearing and prominent, sometimes multiple nucleoli are present. The cytoplasm is often abundant, but may be scant or moderate and poorly defined, unless stripped. A characteristic tigroid (stripped) background is typically present on May‐Grümwald‐Giemsa stained smears. Mitotic figures may be observed. Lymphocytes, plasma cells and giant cells are observed in the background. Cytological features of seminoma are shown in Figure 4. Thymoma and large cell lymphoma represent the principal diagnosis differential in mediastinal EGGCT. FNAC smears of nonseminoma tumors show heterogeneous morphological aspects. Cohesive clusters or acinar structures constituted of cells with vacuolated cytoplasm and large nuclei are observed in YST. Extracellular matrix and hyaline globules may be also observed. Choriocarcinoma is characterized by giant multinucleated tumor cells. Numerous anaplastic isolated cells or hyperchromatic nuclei with nucleoli and scant, poorly defined basophilic cytoplasm arranged in glandular or papillary structures are observed in EC. Mitotic figures are frequent. Necrosis and hemorrhage may be observed on cytological smears. The diagnosis of teratoma could be suspected when squamous cells, columnar cells and mesenchymal portions are observed. Immature forms may be more difficult to diagnose on a cytological sample as well as mixed forms. Metastatic neoplasms of the lung or gastroenteric tract and melanoma represent their differential diagnosis.^33^, ^34^, ^35^

## 6 IMMUNOHISTOCHEMISTRY

Immunohistochemistry (IHC) is very helpful in order to establish the diagnosis of different components occurring in EGGCT. The most useful IHC markers are listed in Table 1. Seminoma expresses several factors of pluripotency regulation also expressed by normal pluripotent germ cell tumors and normal gonocytes, such as PLAP, POU5F1(OCT4), NANOG, SOX2, REX1, UTF1, KIT (CD117) or LIN28.^36^, ^37^, ^38^, ^39^, ^40^, ^41^, ^42^, ^43^, ^44^, ^45^ Some of these embryonic factors, like NANOG and oct4, are also expressed by EC; however, CD30 and SOX2 are more specific markers for diagnosis of EC, allowing differential diagnosis with seminoma.^46^YST reproduces the immunophenotype of human yolk sac and early endoderm, and consequently expresses alpha fetoprotein (αFP), Glypican‐3, Villin, SALL4 and LIN28.^47^ However, the most informative markers for YST commonly used in clinical practice are αFP, Glypican‐3 and SALL4.^48^ ZBTB16 (Zinc finger and BTB domain‐containing protein 16) is a factor involved in cell cycle progression, recently proposed as possible diagnostic marker for YST. Indeed ZBTB16 is highly sensitive and specific for YST, as it is expressed in 91.6% of extragonadal and metastatic YST.^49^ Cytokeratins are expressed by nonseminomatous GCTs and may have a diagnostic role in differentiating seminoma from nonseminomatous mimickers like EC and YSTs. Nevertheless, a dot‐like positivity of low molecular weight cytokeratin has been demonstrated in almost 80% of mediastinal seminomas.^50^ beta human chorionic gonadotropin (β‐hCG) is the most sensitive marker for choriocarcinoma, which also expresses Glypican‐3 and SALL4.^51^ The diagnosis of teratoma generally does not require IHC, but it could be needed in the case of suspect immature component. Indeed S100 and synaptophysin are observed in neural components, whilst desmin and myogenin in muscle components and S100 and in cartilagineous components.

**Table 1:** Immunohistochemical features of EGGCTs

Histotype	CK	αFP	βhCG	CD117	PLAP	CD30	OCT4	SALL4	Glypican 3	NANOG	LIN28	SOX2
SE	Neg^a^	Neg	Neg^b^	Pos	Pos	Neg	Pos	Pos	Neg	Pos	Pos	Neg
EC	Pos	Neg/Pos	Neg^b^	Neg	Neg	Pos	Pos	Pos	Neg/Pos	Pos	Pos/Neg	Pos
YSC	Pos	Pos	Neg^b^	Neg	Neg/Pos	Neg/Pos	Neg	Pos	Pos	Neg	Neg	Neg
PT	Pos	Neg	Neg	Neg	Neg	Neg	Neg	Neg	Neg	Neg	Neg	Neg
CHC	Pos	Neg	Pos	Neg	Neg	Neg	Neg	Pos^c^	Pos	Neg	Neg	Neg/Pos

Abbreviations: αFP, alpha fetoprotein; β‐hCG, beta human chorionic gonadotropin; CHC, choriocarcinoma; EC, embryonal carcinoma; PT, pure teratoma; SE, seminoma; YSC, yolk sac tumor.

a Dot‐like positivity can be observed.

b Syncytiotrophoblastic cells are positive, if present.

c Positive in mononuclear trophoblastic cells.

## 7 SERUM MARKERS

The diagnostic process of GCTs requires the measure of specific biochemical serum tumor markers (STMs). STMs results frequently elevated in EGGCTs and are helpful for both diagnosis and follow‐up. The most commonly increased STMs in EGGCT patients include αFP, β‐hCG and lactate dehydrogenase (LDH). Particularly, αFP is frequently increased in nonseminomatous EGGCTs (proportionally higher according to the disease stage), while it is never increased in pure seminoma patients.^52^ Serum αFP is almost always increased in pure YST or containing YST‐mixed GCT patients. Serum β‐hCG may be increased in pure seminoma,and nonseminomatous GCTs, in both cases more frequently in advanced disease.^52^ The presence of multinuclear trophoblastic‐like giant cells in a seminoma is related to β‐hCG production.^53^ Serum LDH, the less specific STM, increases in 40%‐60% of GCTs patients regardless of the histological type. Serum LDH may reach higher levels when a relevant gain of chromosome 12p is present, being *LDHB* gene located on 12p.^54^ STMs are recommended by the International Germ Cell Cancer Collaborative Group system to monitor the treatment and stratify the risk of nonseminomatous GSTs patients but not for seminoma patients.^55^ The interpretation of these “classical” serum markers is often difficult, because several different clinical conditions could affect their serum increase. Most recently, some micro‐RNA profiles (miRNAs) as in the embryonic stem cells have been linked to GCTs. These miRNAs types seem to be highly upregulated and detectable in the serum of GCT patients, regardless of age and sex of patients.^56^ Preliminary studies concerning the detection of miR‐371‐3 cluster, miR‐302 and miR‐367, showed great specificity (99%‐100%) and sensitivity in GCTs other than pure teratoma.^57^, ^58^, ^59^, ^60^ Furthermore, miRNAs have also been detected in pleural effusions and cerebrospinal fluid, in patients with GCTs.^60^, ^61^ Although the diagnostic utility of miRNAs looks very promising, these preliminary studies considered only testicular GCTs and further exploration is mandatory.

## 8 PROGNOSIS

Prognosis is strictly related to the patient age, histological type, and anatomic location. The latter affects the prognosis for the direct effects of the tumor on vital organs in such site.^11^ Although most neonatal and congenital extragonadal teratomas are benign, about 10% of congenital sacrococcygeal teratomas have a malignant behavior.^62^, ^63^ Congenital and neonatal extragonadal teratomas are usually immature and a YST component is often associated. Although the presence of admixed YST has unclear clinical significance, neonatal mixed teratomas seem to have a poor prognosis.^64^ In congenital and neonatal extragonadal teratomas the presence of immature neuroepithelium is not predictive per se of malignant behavior, but immature teratomas are more likely associated to admixed YST component.^20^ Other factors associated to the presence of admixed YST include relatively older patients and sacrococcygeal location.^11^ Hemorrhage is the most common lethal complication in neonatal patients with sacrococcygeal teratomas.^65^ Increasing age negatively affects the clinical behavior and the prognosis of completely resected EGGCTs, worsening at approximately 7 months.^20^ Histotype is the most important prognostic factor in adult EGGCTs, being seminomas long‐time survival rate of about 90% and nonseminomatous of about 45%.^31^ The presence of immature neuroepithelium in adult extragonadal teratomas has an important prognostic role.^66^, ^67^, ^68^, ^69^ Histologically, mature teratomas have a benign behavior regardless of patient's age; immature teratomas behave as aggressive tumors in adults. In EGGCTs adult patients, clinical and pathological staging, primary location in the mediastinum, increased serum β‐hCG and nonpulmonary visceral metastases are further independent prognostic factors related to shorter survival.^31^

## 9 CLINICAL MANAGEMENT

In the past, complete surgical resection of the tumoral masses was the standard therapy for EGGCTs patients. But EGGCTs, particularly mediastinal EGGCTs and EGGCTs including NST components, related with a poorer prognosis when treated with surgical resection alone. Recently, the introduction of cisplatin‐based chemotherapy in these clinical settings as in gonadal counterpart has dramatically improved the prognosis.^70^, ^71^, ^72^

Nonetheless, in several EGGCT patients a persistent residual mass after cisplatin‐based chemotherapy and conventional selvage chemotherapy could be observed. Thus, the residual mass resection after chemotherapy is necessary in order to obtain radical control of EGGCt morbidity. The presence of residual tumor is often due to the unresponsiveness of teratoma components in EGGCTs mass.^15^ Therefore, this multidimensional approach is needed to assess response, to remove residual disease and eventually to turn to additional chemotherapy.^73^

In the case of localized EGGCTs, patients can really benefit from radical surgery and subsequent chemiotherapy. Indeed, surgical excision demonstrated advantages in order of disease free‐survival, when complete excision can be performed.^74^

## 10 CONCLUSION

In conclusion, EGGCTs represent a rare group of neoplasms, occurring more commonly in the mediastinum and retroperitoneum. The EGGCTs are histologically constituted by the same components observed in the gonadal counterparts, but they are characterized by different biologic behaviors, clinical features and poorer prognoses, mainly when constituted by nonsemonomatous components. The efficacy of multidimensional therapy, frequently practised in mediastinal EGGCTs, depends on both successful chemotherapy and surgery. Currently new therapeutic strategies are being studied to improve the prognosis, in order to obtain a longer overall survival, similar to that observed in the gonadal germ cell tumors.

## CONFLICT OF INTEREST

All the authors declare no conflict of interest.
